# Effects of Exergames on Brain Dynamics in Women with Fibromyalgia: A Randomized Controlled Trial

**DOI:** 10.3390/jcm8071015

**Published:** 2019-07-11

**Authors:** Santos Villafaina, Daniel Collado-Mateo, Juan Pedro Fuentes, Paloma Rohlfs-Domínguez, Narcís Gusi

**Affiliations:** 1 Physical Activity and Quality of Life Research Group (AFYCAV), Faculty of Sport Sciences, University of Extremadura, 10003 Cáceres, Spain; 2 Education Faculty, Universidad Autonoma de Chile, Talca 1670, Chile; 3 Faculty of Sport Sciences, University of Extremadura, 10003 Cáceres, Spain; 4 Physical Activity and Quality of Life research group (AFYCAV), Department of Psychology and Anthropology, University of Extremadura, 10003 Cáceres, Spain; 5 Department of Social Psychology and Methodology of Behavior, University of Basque Country-Euskalherriko Univertsitatea, 48940 Leioa (Bilbao), Spain; 6 CIBER de Fragilidad y Envejecimiento Saludable, Av. Monforte de Lemos, 28029 Madrid, Spain

**Keywords:** virtual reality, chronic pain, EEG, exercise, brain

## Abstract

Background: Exergames are non-immersive versions of virtual reality that involve physical exercise and have shown several benefits on physical fitness and quality of life in women with fibromyalgia. However, the effects on brain dynamics are still unknown. Aim: the aim was to evaluate the effects of a 24-week exergame intervention on resting brain dynamics in women with fibromyalgia in a single-blinded, randomized controlled trial. Methods: Fifty-six women with fibromyalgia were assessed for eligibility; 55 fulfilled the inclusion criteria. The exercise group completed a 24-week exergame-based intervention that focused on mobility, postural control, upper and lower limb coordination, aerobic fitness, and strength. This group received two 60-min sessions per week. We measured electroencephalographic (EEG) signals from 19 channels. Participants were also divided into two subgroups according to the duration of their symptoms. The intervention was more effective in the group with a shorter duration of symptoms, showing between-group differences in F8, T5 and T4. Conclusion: Exergames may lead to changes in brain dynamics that could be related to increased cerebral blood flow.

## 1. Introduction

Fibromyalgia is a chronic condition characterized by widespread musculoskeletal pain accompanied by fatigue, stiffness, sleep disturbance, and cognitive impairments [1] in areas including memory [2], processing speed [3], cognitive flexibility [4], and decision making [5]. Fibromyalgia leads to a reduction in the ability to perform activities of daily life [6] and in the quality of life [7]. Electroencephalographic (EEG) studies have reported abnormal dynamics in the brain at rest [8,9,10,11], while eliciting depression symptoms [12] and during pain processing [13,14,15] in patients with fibromyalgia.

Of all non-pharmacological therapies, physical exercise has the highest level of evidence for reducing fibromyalgia symptoms [16]. Prior neuroscientific research has examined the effects of exercise-based interventions on brain activation in patients with fibromyalgia [17,18]. In particular, studies show that physical exercise programs may normalize cognitive processing, reflected by increased task-related activation of the amygdala [17], and the abnormal resting state functional connectivity [18].

Animal studies indicate that some of the effects of physical exercise on cognitive function are related to neurally driven angiogenesis in the paramedian lobule of the cerebellum [19,20,21]. In this regard, an EEG study showed that a decrease in cerebral blood flow lead to a decrease in power in the EEG beta frequency band [22]. Therefore, one would anticipate the opposite, i.e., an increase in beta-band power, with the increased cerebral blood flow derived from exercise [22,23].

Virtual reality (VR) programs have emerged as an effective form of therapy in a variety of patient populations [24,25,26]. Exergames, non-immersive versions of VR that involve physical exercise [27], have previously been used in patients with fibromyalgia, resulting in improvements in mobility and the overall quality of life [28,29]. However, the effects of exergame-based interventions on the brain dynamics of patients with fibromyalgia have not previously been studied. Therefore, this study aims to evaluate the effects of an exergame-based intervention on the brain dynamics of women with fibromyalgia via analysis of the EEG power spectrum. Since exercise-based interventions lead to angiogenesis in the brain and changes in cerebral blood flow can be detected as changes in the EEG beta power, we hypothesized that participants would show increased power in the beta frequency band after the exergame intervention.

## 2. Experimental Section

### 2.1. Participants

The sample size was determined on the basis of the fibromyalgia impact questionnaire (FIQ) score [30], where a reduction of 14% is considered to be clinically important [31]. Previous Spanish studies in fibromyalgia patients indicate that a mean FIQ score of 70.5 (11.8) is expected prior to intervention [32]. To detect significant between-group differences of at least 14% with an α value of 0.05 and power of 85%, we needed to enroll a minimum of 26 participants per group. Ultimately, 56 participants recruited by the local fibromyalgia association until Dec 31, 2017, fulfilled the following inclusion criteria:

- Female and aged between 30 and 75 years.
- Able to communicate with the research staff.
- Able to read and sign the written informed consent.
- Diagnosed with fibromyalgia by a rheumatologist according to the criteria of the American College of Rheumatology [1].

Participants were excluded if they:

- Changed their usual care therapies during the 24 weeks of the treatment.
- Had contraindications for physical exercise programs and/or were pregnant.

One participant was excluded for not meeting the inclusion criteria, so the final participant sample consisted of 55 participants. All the participants were randomly assigned to the experimental group (EG; n = 28) or the control group (CG; n = 27) via random numbers. This process was done by one researcher who did not participate in the data acquisition or statistical analysis. Both the pre- and post-evaluations were conducted by a researcher who was blinded to the grouping allocation. However, the participants themselves were not blinded to their treatment group because they had to read and sign the written informed consent, which included all the procedures.

**Figure 1:**
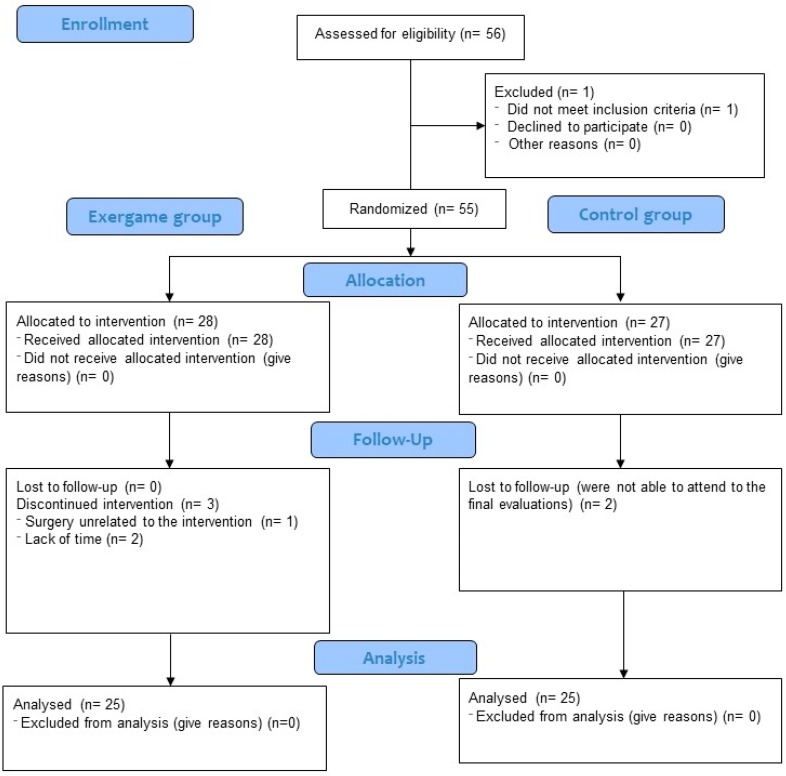
Flow chart of participants.

During the exergame-based intervention, three participants (10.7%) were lost to follow-up because they did not fulfill the minimum 75% attendance rate. Moreover, two participants (7.41%) from the CG did not come to the final evaluation session. The flow chart of participants is shown in Figure 1.

Baseline characteristics are reported in Table 1. There were no significant differences at baseline for the following variables: medication intake, age, mean of number of years with fibromyalgia symptoms, or impact of fibromyalgia as assessed with the Fibromyalgia Impact Questionnaire (FIQ).

### 2.2. Experimental Design

The sample (n = 55) for this single-blinded randomized controlled trial was divided into two groups (EG and CG). The protocol for the trial is available at the following website: https://doi.org/10.1186/ISRCTN65034180. The trial was prospectively registered at the International Standard Randomised Controlled Trial Number Registry (ISRCTN65034180). The University research ethics committee approved all the procedures (62/2017).

However, a few changes were made to the original protocol: the sample size was increased to obtain higher statistical power, and the intervention was completed entirely at the university facilities.

The current study focuses on the effects of exergames on brain dynamics, which is one of the five primary outcomes of the trial. Two of the five primary outcomes, focused on quality of life and physical fitness, have been recently published elsewhere [33,34]. This is justified given that the hypothesis in the current study is entirely novel and different from the previous studies, and also the research field (neuroscience) is completely different from that for the other primary variables, involving other specific research professionals and audience.

### 2.3. Intervention

The EG completed 24 weeks of an exergame-based intervention while the CG continued with their usual care and daily life, free of alteration. The exergame-based intervention was conducted at the university facilities, where participants completed two sessions per week (1 h per session) in groups of two or three participants.

The exergame (VirtualEx-FM) was created by the research group with the goal of improving the ability to perform daily life activities. VirtualEx-FM fulfills all the criteria suggested by Lewis and Rosie [35], which lists eight key points for VR rehabilitation therapy tools. Moreover, previous studies using VirtualEx-FM reported improvements in mobility skills and quality of life [28,29]. A typical VirtualEx-FM session consisted of the following components:

Warm-up: joint movements guided by a pre-recorded video of a kinesiology professional.

Aerobic component: dance steps guided by a video of a dance teacher.

Postural control and coordination games: participants had to reach for an apple that appeared and disappeared in different locations near them. The body part that participants had to use to reach the apple was indicated by the application and could be manually controlled by the kinesiology professional.

Walking training: participants had to follow a virtual trail of footprints. Different types of steps (normal, tiptoe, heel walking, raised heels, and raised knees) were allowed. No harm was observed along the intervention.

### 2.4. Outcome Measures

EEG signals were assessed pre- (one week before starting the intervention) and post-exergame intervention (one week after ending the intervention) with an Enobio device (Neuroelectrics, Cambridge, MA, USA) [36] during a 1-min resting period with eyes closed. This procedure was also followed in previous studies with women with fibromyalgia [10,11]. The wireless Enobio electrode system is reliable even with dry electrodes [37]. EEG signals were recorded from 19 locations according to the International 10–20 system: frontal (Fz, Fp1, Fp2, F3, F4, F7 and F8), central (Cz, C3, and C4), temporal (T3, T4, T5 and T6), parietal (Pz, P3, and P4), and occipital (O1 and O2). Both, CG and EG groups were evaluated in the same period of time.

Electrodes placed in the mastoids served as references and impedance was kept below 10 KΩ. EEG was recorded at a sampling rate of 500 Hz with bandpass filtering (1–40 Hz) and a 50 Hz notch filter. The Matlab EEGlab toolbox was employed for pre-processing the data and data analysis.

Epochs with rough artifacts were manually removed and non-cortical sources (eye-movements, muscle activity, line noise, etc.) were corrected using independent component analysis (ICA) [38]. During recordings, participants were seated on a chair in a calm room and were encouraged to keep their eyes closed to minimize artifacts.

After pre-processing, the EEGlab function pop_spectopo.m was used to separate the data into theta (4–7 Hz), alpha-1 (8–10 Hz), alpha-2 (11–12 Hz), beta-1 (13–18 Hz), beta-2 (19–21 Hz), and beta-3 (22–30 Hz) frequency bands.

### 2.5. Statistical Analysis

In light of the results obtained from the Shapiro–Wilk and Kolmogorov–Smirnov tests, the data were analyzed with non-parametric methods.

The Chi-squared and Mann–Whitney U tests were used to assess between-group differences in medication intake, age, number of years with fibromyalgia symptoms and fibromyalgia impact as well as in the EEG-frequency spectrum bands at baseline. The Mann–Whitney U test was used to assess between-group differences for the different frequency bands. Therefore, EEG-frequency bands values were adjusted to baseline in order to compare the effect of the intervention.

Additional sub- and between-group analyses were also conducted. In particular, the median number of years that participants had suffered from fibromyalgia symptoms was used as a threshold to divide the sample into two subgroups (shorter and longer duration of fibromyalgia symptoms). This enabled us to explore whether the duration of fibromyalgia symptoms altered the effectiveness of the exergame-based intervention. This strategy for splitting sub-groups of patients was previously performed in women with fibromyalgia [10,39].

Data from all 55 initial participants were used to conduct the intention-to-treat analysis by multiple imputation (MI) of missing values following the Sterne et al. [40] guidelines. Our missing data were classified as missing at random.

The alpha-level of statistical significance (set at 0.05) was adjusted according to the Benjamini-Hochberg procedure to control the false discovery rate [41].

The SPSS statistical package (version 20.0; SPSS, Inc., Chicago, IL, USA) was used to perform the analyses.

## 3. Results

Differences between groups were not observed in any of the studied frequency bands at baseline. 

**Figure 2:**
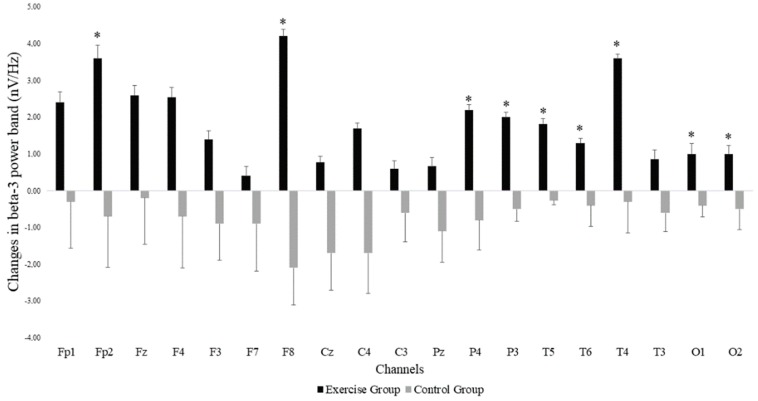
Changes in beta-3 band power at the different EEG electrode locations in both exergame and control groups. Error bars represent the standard error of the mean. *: Significant differences (*p* < 0.05) between the exercise and the control groups after the exergames intervention.

The Mann–Whitney U test showed that there was a significant group*time interaction for power in the beta-3 frequency band across the different EEG electrode locations, with all changes in favor of the EG. Significant differences were found in the frontal (Fp2: *p*-value = 0.037 and F8: *p*-value = 0.002) parietal (P4: *p*-value = 0.014 and P3: *p*-value = 0.031), temporal (T6: *p*-value = 0.037, T5: *p*-value = 0.001 and T4: *p*-value = 0.040) and occipital (O1: *p*-value = 0.040 and O2: *p*-value = 0.035) areas. Figure 2 shows the EEG locations where differences were observed. In addition to increases in beta-3 band power in the EG, we observed decreases in beta-3 band power in the CG. Non-significant effects were found in the remaining EEG-frequency bands.

Participants were divided into two subgroups according to the number of years they had suffered from fibromyalgia symptoms. The median duration of fibromyalgia symptoms (17 years) was used as the threshold to divide the participants into a short-duration group (EG: n = 15 and CG: n = 15) and a long-duration group (EG: n = 13 and CG: n = 10). Two CG participants were excluded from this analysis because they could not remember how long they had suffered from fibromyalgia. The two duration subgroups were analyzed separately with group (EG and CG) and time (pre- and post- intervention) as the variables.

**Figure 3:**
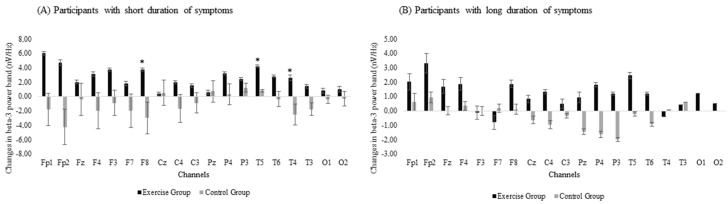
Changes in beta-3 band power for subgroups with (**A**) short duration or (**B**) long duration of symptoms. Error bars represent the standard error of the mean. *: Significant differences (*p* < 0.05) between the exercise and the control groups after the exergames intervention.

The subgroup analysis revealed between-group differences in the effectiveness of the exergame-based program when the duration of symptoms was included in the analysis, with differences between the EG and the CG detected in the subgroup that had suffered from fibromyalgia-related symptoms for fewer years. There were significant group × time interactions in beta-3 band power in the frontal (F8: *p*-value = 0.008) and temporal (T5: *p*-value = 0.004 and T4: *p*-value = 0.014) areas (Figure 3A). However, no significant differences were observed for the long-duration subgroup (Figure 3B).

## 4. Discussion

Here, we demonstrate that an exergame-based intervention in participants with fibromyalgia increased beta-3 band power in frontal, parietal, temporal and occipital areas. Moreover, the effectiveness of the exergames intervention on changes in beta-3 band power depended on the length of time that patients had suffered from fibromyalgia symptoms. Specifically, the exergames-based intervention may only be effective in patients who have had fibromyalgia-related symptoms for a shorter period of time (<17 years). Exergames have been used previously to improve both physical function and quality of life in women with fibromyalgia after 8-week [28,29] and 24-week interventions [33,34]. However, this is, to our knowledge, the first study to report a significant effect of an exergame-based intervention on brain dynamics in patients with fibromyalgia.

Animal studies indicate that some of the benefits of exercise may be mediated by the enhancement of angiogenesis and neurogenesis [19,20,21]. Furthermore, in humans it is known that early exercise after a stroke can improve cerebral blood flow through increased angiogenesis [42]. Therefore, the effects of regular exercise on the brain are of great interest to the field of neuroscience. In our study, we observed increased power in the beta-3 frequency band after the exergame-based intervention. Reduced cerebral blood flow (because of anoxia or hypoxia) is associated with a decrease in cortical beta-band power [22]. Our finding of an increase in beta-band power with exercise is consistent with this given that physical exercise promotes cerebral blood flow [43]. In other words, exercise has the opposite effect to anoxia or hypoxia [23,44], and leads to an increase in the beta-band power during brain reoxygenation [45]. Our results also agree with a previous study that examined the potential changes in the EEG signals as a result of long-term physical exercise [23]. Participants with a high level of fitness (enrolled in sports or with a minimum three-year history of vigorous aerobic physical exercise) showed increased power in the beta band that may have been due to increased cerebral blood flow [23].

In addition, lower cerebral blood flow is related to poor memory functioning [46,47], and exercise training can increase cerebral blood flow velocity, leading to a beneficial effect on cognitive function [48,49,50]. These results are particularly relevant to patients with fibromyalgia since such patients frequently have altered cerebral blood flow variability [51] and velocity [52], as well as impaired cognitive function [53] such as memory [54] or poor performance in tests of executive functions [55]. Therefore, exergame interventions in fibromyalgia could increase the cerebral blood flow and, in consequence, the cognitive function. Interestingly, our study showed that control group decreased the EEG beta-3 power. This results could be expected since middle-aged and elderly adults may experience a decrease of 0.45% to 0.50% in global cerebral blood flow per year [56,57,58]. Therefore, further research is needed to examine the potential correlations between the improvements in EEG beta-3 band power and changes in cerebral blood flow, and how this interaction may influence common cognitive impairments in patients with fibromyalgia.

In the present study, we compared the effectiveness of the exergames-based intervention in two subgroups of patients who had suffered from fibromyalgia-related symptoms for different lengths of time. Given the degree of heterogeneity that is typically seen in patients with fibromyalgia [59], subgroup analyses are strongly recommended to examine the effectiveness of the intervention as a function of the individual patient profiles [59,60]. Our subgroup analysis found significant differences in beta-3 band power in frontal and temporal areas, indicating that the effectiveness of the exergame-based intervention was greater in the group of patients who had experienced fibromyalgia-related symptoms for a shorter period of time. These results are relevant because previous research has shown that a longer duration of fibromyalgia-related symptoms predicts a lower FIQ score, thus indicating that participants who have experienced fibromyalgia-related symptoms for shorter periods of time may be more severely affected by the disease [61]. In the present study, we also observed a similar inverse correlation between the duration of fibromyalgia-related symptoms and the impact of the disease (Spearman’s Rho = −0.295; *p*-value = 0.032). These results suggest that exergames may counteract the potential decrease in EEG beta-3 band power that manifests with severe fibromyalgia-related symptoms.

The present study has some limitations that should be considered. First, we did not include a participant group performing a traditional exercise program to serve as an active CG. This active CG would have enabled us to isolate the effect of the targeted exergame-based intervention. Second, we included only female patients with fibromyalgia. Therefore, we cannot generalize the reported effects to male patients with fibromyalgia. Third, the relatively small sample size likely reduced the statistical significance of some of our results; it is possible that only the largest differences had enough statistical power to reach significance.

## 5. Conclusions

This study demonstrates that exergames may be a useful tool to improve EEG beta-band power in patients with fibromyalgia. These beta-band enhancements may be related to increased cerebral blood flow. Moreover, the effectiveness of the exergame-based intervention appears to be greater in patients who have had fibromyalgia-related symptoms for a shorter period of time.

## Figures and Tables

**Table 1:** Demographic data at baseline.

Variable	Exercise Group Median (IQR)	Control Group Median (IQR)	Value of Contrast	*p*-Value ^a^
Sample size	28	27		
Age (years)	52.00 (17)	54.00 (13)	−0.110	0.913
FIQ-r total score	53.08 (30.5)	60.83 (29.33)	−0.025	0.980
Years with chronic pain	16.00 (11.8)	16.00 (14.5)	−0.420	0.674
≤10 (frequency and %)	6 (21.4%)	7 (28%)		
<10–20 (frequency and %)	10 (35.7%)	9 (32%)		
≥20 (frequency and %)	12 (42.8%)	11 (40%)		

^a^*p*-value of Mann–Whitney U tests. IQR: Interquartile range; FIQ: Fibromyalgia impact questionnaire.

## References

[1] Wolfe F., Clauw D.J., Fitzcharles M.A., Goldenberg D.L., Katz R.S., Mease P., Russell A.S., Russell I.J., Winfield J.B., Yunus M.B. (2010). The American College of Rheumatology Preliminary Diagnostic Criteria for Fibromyalgia and Measurement of Symptom Severity. Arthritis Care Res..

[2] Duschek S., Werner N.S., Winkelmann A., Wankner S. (2013). Implicit memory function in fibromyalgia syndrome. Behav. Med..

[3] Reyes Del Paso G.A., Montoro C.I., Duschek S. (2015). Reaction time, cerebral blood flow, and heart rate responses in fibromyalgia: Evidence of alterations in attentional control. J. Clin. Exp. Neuropsychol..

[4] Gelonch O., Garolera M., Valls J., Rossello L., Pifarre J. (2016). Executive function in fibromyalgia: Comparing subjective and objective measures. Compr. Psychiatry.

[5] Walteros C., Sanchez-Navarro J.P., Munoz M.A., Martinez-Selva J.M., Chialvo D., Montoya P. (2011). Altered associative learning and emotional decision making in fibromyalgia. J. Psychosom. Res..

[6] Huijnen I.P.J., Verbunt J.A., Meeus M., Smeets R. (2015). Energy Expenditure during Functional Daily Life Performances in Patients with Fibromyalgia. Pain Pract..

[7] Burckhardt C.S., Clark S.R., Bennett R.M. (1993). Fibromyalgia and quality of life: A comparative analysis. J. Rheumatol..

[8] Hargrove J.B., Bennett R.M., Simons D.G., Smith S.J., Nagpal S., Deering D.E. (2010). Quantitative Electroencephalographic Abnormalities in Fibromyalgia Patients. Clin. EEG Neurosci..

[9] Gonzalez-Roldan A.M., Cifre I., Sitges C., Montoya P. (2016). Altered Dynamic of EEG Oscillations in Fibromyalgia Patients at Rest. Pain Med..

[10] Villafaina S., Collado-Mateo D., Fuentes-García J.P., Domínguez-Muñoz F.J., Gusi N. (2019). Duration of the Symptoms and Brain Aging in Women with Fibromyalgia: A Cross-Sectional Study. Appl. Sci..

[11] Villafaina S., Collado-Mateo D., Fuentes-García J.P., Cano-Plasencia R., Gusi N. (2019). Impact of Fibromyalgia on Alpha-2 EEG Power Spectrum in the Resting Condition: A Descriptive Correlational Study. BioMed Res. Int..

[12] Villafaina S., Sitges C., Collado-Mateo D., Fuentes-García J.P., Gusi N. (2019). Influence of depressive feelings in the brain processing of women with fibromyalgia: An EEG study. Medicine.

[13] Staud R., Craggs J.G., Perlstein W.M., Robinson M.E., Price D.D. (2008). Brain activity associated with slow temporal summation of C-fiber evoked pain in fibromyalgia patients and healthy controls. Eur. J. Pain.

[14] Gracely R.H., Petzke F., Wolf J.M., Clauw D.J. (2002). Functional magnetic resonance imaging evidence of augmented pain processing in fibromyalgia. Arthritis Rheum..

[15] Burgmer M., Pogatzki-Zahn E., Gaubitz M., Wessoleck E., Heuft G., Pfleiderer B. (2009). Altered brain activity during pain processing in fibromyalgia. Neuroimage.

[16] Bidonde J., Jean Busch A., Bath B., Milosavljevic S. (2014). Exercise for adults with fibromyalgia: an umbrella systematic review with synthesis of best evidence. Curr. Rheum. Rev..

[17] Martinsen S., Flodin P., Berrebi J., Lofgren M., Bileviciute-Ljungar I., Mannerkorpi K., Ingvar M., Fransson P., Kosek E. (2018). The role of long-term physical exercise on performance and brain activation during the Stroop colour word task in fibromyalgia patients. Clin. Physiol. Funct. Imaging.

[18] Flodin P., Martinsen S., Mannerkorpi K., Löfgren M., Bileviciute-Ljungar I., Kosek E., Fransson P. (2015). Normalization of aberrant resting state functional connectivity in fibromyalgia patients following a three month physical exercise therapy. NeuroImage Clin..

[19] Black J.E., Isaacs K.R., Anderson B.J., Alcantara A.A., Greenough W.T. (1990). Learning causes synaptogenesis, whereas motor activity causes angiogenesis, in cerebellar cortex of adult rats. Proc. Natl. Acad. Sci. USA.

[20] Kleim J.A., Cooper N.R., VandenBerg P.M. (2002). Exercise induces angiogenesis but does not alter movement representations within rat motor cortex. Brain Res..

[21] Swain R.A., Harris A.B., Wiener E.C., Dutka M.V., Morris H.D., Theien B.E., Konda S., Engberg K., Lauterbur P.C., Greenough W.T. (2003). Prolonged exercise induces angiogenesis and increases cerebral blood volume in primary motor cortex of the rat. Neuroscience.

[22] Kraaier V., Van Huffelen A.C., Wieneke G.H., Van der Worp H.B., Bar P.R. (1992). Quantitative EEG changes due to cerebral vasoconstriction. Indomethacin versus hyperventilation-induced reduction in cerebral blood flow in normal subjects. Electroencephalogr. Clin. Neurophysiol..

[23] Lardon M.T., Polich J. (1996). EEG changes from long-term physical exercise. Biol. Psychol..

[24] Adamovich S.V., Fluet G.G., Tunik E., Merians A.S. (2009). Sensorimotor training in virtual reality: A review. NeuroRehabilitation.

[25] Jansen-Kosterink S.M., Huis In ’t Veld R.M., Schonauer C., Kaufmann H., Hermens H.J., Vollenbroek-Hutten M.M. (2013). A Serious Exergame for Patients Suffering from Chronic Musculoskeletal Back and Neck Pain: A Pilot Study. Games Health J..

[26] Park E.C., Kim S.G., Lee C.W. (2015). The effects of virtual reality game exercise on balance and gait of the elderly. J. Phys. Ther. Sci..

[27] Wuest S., van de Langenberg R., de Bruin E.D. (2014). Design considerations for a theory-driven exergame-based rehabilitation program to improve walking of persons with stroke. Eur. Rev. Aging Phys. Act. Off. J. Eur. Group Res. Elder. Phys. Act..

[28] Collado-Mateo D., Dominguez-Munoz F.J., Adsuar J.C., Garcia-Gordillo M.A., Gusi N. (2017). Effects of Exergames on Quality of Life, Pain, and Disease Effect in Women With Fibromyalgia: A Randomized Controlled Trial. Arch. Phys. Med. Rehabil..

[29] Collado-Mateo D., Dominguez-Muñoz F.J., Adsuar J.C., Merellano-Navarro E., Gusi N. (2017). Exergames for women with fibromyalgia: A randomised controlled trial to evaluate the effects on mobility skills, balance and fear of falling. PeerJ.

[30] Bennett R. (2005). The Fibromyalgia Impact Questionnaire (FIQ): A review of its development, current version, operating characteristics and uses. Clin. Exp. Rheumatol..

[31] Bennett R.M., Bushmakin A.G., Cappelleri J.C., Zlateva G., Sadosky A.B. (2009). Minimal clinically important difference in the fibromyalgia impact questionnaire. J. Rheumatol..

[32] Esteve-Vives J., Rivera Redondo J., Isabel Salvat Salvat M., de Gracia Blanco M., de Miquel C.A. (2007). [Proposal for a consensus version of the Fibromyalgia Impact Questionnaire (FIQ) for the Spanish population]. Reumatol. Clin..

[33] Villafaina S., Collado-Mateo D., Dominguez-Munoz F.J., Fuentes-Garcia J.P., Gusi N. (2019). Benefits of 24-Week Exergame Intervention on Health-Related Quality of Life and Pain in Women with Fibromyalgia: A Single-Blind, Randomized Controlled Trial. Games Health J..

[34] Martin-Martinez J.P., Villafaina S., Collado-Mateo D., Perez-Gomez J., Gusi N. (2019). Effects of 24-wk exergame intervention on physical function under single- and dual-task conditions in fibromyalgia: A randomized controlled trial. Scand. J. Med. Sci. Sports.

[35] Lewis G.N., Rosie J.A. (2012). Virtual reality games for movement rehabilitation in neurological conditions: how do we meet the needs and expectations of the users?. Disabil. Rehabil..

[36] Ruffini G., Dunne S., Farres E., Cester I., Watts P.C.P., Silva S.R.P., Grau C., Fuentemilla L., Marco-Pallares J., Vandecasteele B. ENOBIO dry electrophysiology electrode; first human trial plus wireless electrode system. Proceedings of the 2007 29th Annual International Conference of the IEEE Engineering in Medicine and Biology Society.

[37] Collado-Mateo D., Adsuar J.C., Olivares P.R., Cano-Plasencia R., Gusi N. (2015). Using a dry electrode EEG device during balance tasks in healthy young-adult males: Test-retest reliability analysis. Somatosens. Motor Res..

[38] Jung T.P., Makeig S., Westerfield M., Townsend J., Courchesne E., Sejnowski T.J. (2000). Removal of eye activity artifacts from visual event-related potentials in normal and clinical subjects. Clin. Neurophysiol..

[39] Amris K., Luta G., Christensen R., Danneskiold-Samsoe B., Bliddal H., Waehrens E.E. (2016). Predictors of improvement in observed functional ability in patients with fibromyalgia as an outcome of rehabilitation. J. Rehabil. Med..

[40] Sterne J.A., White I.R., Carlin J.B., Spratt M., Royston P., Kenward M.G., Wood A.M., Carpenter J.R. (2009). Multiple imputation for missing data in epidemiological and clinical research: Potential and pitfalls. BMJ (Clin. Res. Ed.).

[41] Benjamini Y., Hochberg Y. (1995). Controlling the false discovery rate: a practical and powerful approach to multiple testing. J. R. Stat. Soc. Ser. B.

[42] Zhang P., Yu H., Zhou N., Zhang J., Wu Y., Zhang Y., Bai Y., Jia J., Zhang Q., Tian S. (2013). Early exercise improves cerebral blood flow through increased angiogenesis in experimental stroke rat model. J. Neuroeng. Rehabil..

[43] Ogoh S., Fadel P.J., Zhang R., Selmer C., Jans O., Secher N.H., Raven P.B. (2005). Middle cerebral artery flow velocity and pulse pressure during dynamic exercise in humans. Am. J. Physiol. Heart Circ. Physiol..

[44] Moraes H., Ferreira C., Deslandes A., Cagy M., Pompeu F., Ribeiro P., Piedade R. (2007). Beta and alpha electroencephalographic activity changes after acute exercise. Arquivos de Neuro-Psiquiatria.

[45] Zhao J.P., Zhang R., Yu Q., Zhang J.X. (2016). Characteristics of EEG activity during high altitude hypoxia and lowland reoxygenation. Brain Res..

[46] Leeuwis A.E., Smith L.A., Melbourne A., Hughes A.D., Richards M., Prins N.D., Sokolska M., Atkinson D., Tillin T., Jäger H.R. (2018). Cerebral Blood Flow and Cognitive Functioning in a Community-Based, Multi-Ethnic Cohort: The SABRE Study. Front. Aging Neurosci..

[47] Birdsill A.C., Carlsson C.M., Willette A.A., Okonkwo O.C., Johnson S.C., Xu G., Oh J.M., Gallagher C.L., Koscik R.L., Jonaitis E.M. (2013). Low cerebral blood flow is associated with lower memory function in metabolic syndrome. Obesity.

[48] Joris P.J., Mensink R.P., Adam T.C., Liu T.T. (2018). Cerebral Blood Flow Measurements in Adults: A Review on the Effects of Dietary Factors and Exercise. Nutrients.

[49] Ainslie P.N., Cotter J.D., George K.P., Lucas S., Murrell C., Shave R., Thomas K.N., Williams M.J., Atkinson G. (2008). Elevation in cerebral blood flow velocity with aerobic fitness throughout healthy human ageing. J. Physiol..

[50] Anazodo U.C., Shoemaker J.K., Suskin N., Ssali T., Wang D.J., St Lawrence K.S. (2015). Impaired Cerebrovascular Function in Coronary Artery Disease Patients and Recovery Following Cardiac Rehabilitation. Front. Aging Neurosci..

[51] Montoro C.I., Duschek S., Schuepbach D., Gandarillas M., Reyes Del Paso G.A. (2018). Cerebral blood flow variability in fibromyalgia syndrome: Relationships with emotional, clinical and functional variables. PLoS ONE.

[52] Rodriguez A., Tembl J., Mesa-Gresa P., Munoz M.A., Montoya P., Rey B. (2017). Altered cerebral blood flow velocity features in fibromyalgia patients in resting-state conditions. PLoS ONE.

[53] Galvez-Sanchez C.M., Reyes Del Paso G.A., Duschek S. (2018). Cognitive Impairments in Fibromyalgia Syndrome: Associations With Positive and Negative Affect, Alexithymia, Pain Catastrophizing and Self-Esteem. Front. Psychol..

[54] Seo J., Kim S.H., Kim Y.T., Song H.J., Lee J.J., Kim S.H., Han S.W., Nam E.J., Kim S.K., Lee H.J. (2012). Working memory impairment in fibromyalgia patients associated with altered frontoparietal memory network. PLoS ONE.

[55] Munoz Ladron de Guevara C., Fernandez-Serrano M.J., Reyes Del Paso G.A., Duschek S. (2018). Executive function impairments in fibromyalgia syndrome: Relevance of clinical variables and body mass index. PLoS ONE.

[56] Leenders K.L., Perani D., Lammertsma A.A., Heather J.D., Buckingham P., Healy M.J., Gibbs J.M., Wise R.J., Hatazawa J., Herold S. (1990). Cerebral blood flow, blood volume and oxygen utilization. Normal values and effect of age. Brain J. Neurol..

[57] Parkes L.M., Rashid W., Chard D.T., Tofts P.S. (2004). Normal cerebral perfusion measurements using arterial spin labeling: reproducibility, stability, and age and gender effects. Magn. Reson. Med..

[58] Zhang N., Gordon M.L., Goldberg T.E. (2017). Cerebral blood flow measured by arterial spin labeling MRI at resting state in normal aging and Alzheimer’s disease. Neurosci. Biobehav. Rev..

[59] Segura-Jiménez V., Soriano-Maldonado A., Álvarez-Gallardo I., Estévez-López F., Carbonell-Baeza A., Delgado-Fernández M. (2016). Subgroups of fibromyalgia patients using the 1990 American College of Rheumatology criteria and the modified 2010 preliminary diagnostic criteria: the al-Ándalus project. Clin. Exp. Rheumatol..

[60] Estévez-López F., Segura-Jiménez V., Álvarez-Gallardo I.C., Borges-Cosic M., Pulido-Martos M., Carbonell-Baeza A., Aparicio V.A., Geenen R., Delgado-Fernández M. (2017). Adaptation profiles comprising objective and subjective measures in fibromyalgia: The al-Ándalus project. Rheumatology.

[61] Van Liew C., Leon G., Neese M., Cronan T.A. (2019). You get used to it, or do you: symptom length predicts less fibromyalgia physical impairment, but only for those with above-average self-efficacy. Psychol. Health Med..

